# Examining the effects of self-regulated learning-based teacher feedback on English-as-a-foreign-language learners’ self-regulated writing strategies and writing performance

**DOI:** 10.3389/fpsyg.2022.1027266

**Published:** 2022-11-08

**Authors:** Li Francoise Yang, Yanhong Liu, Zhiqing Xu

**Affiliations:** ^1^School of Foreign Languages, Jiangsu University of Science and Technology, Zhenjiang, Jiangsu, China; ^2^School of Foreign Studies, Yanshan University, Qinhuangdao, Hebei, China; ^3^School of Foreign Language Education, Jilin University, Changchun, Jilin, China

**Keywords:** second language writing, SRL-based feedback, treatment effects, EFL/L2 writing, SRL writing strategies, writing performance

## Abstract

The current study examines the effect of teacher feedback on fostering self-regulated English-as-a-foreign-language (EFL) writers. Adopting a quasi-experimental design, this study was conducted among seventy students from two parallel intact English writing classes at the tertiary level. While conventional feedback at the level of task was used in the control group, feedback at the level of process and self-regulation with supplementary activities was adopted for the treatment group. This SRL-based feedback intervention lasted one semester. Students took a pre-test, an immediate and a delayed post-test to measure their improvement in English writing performance, as well as their use of writing strategies for self-regulated learning (SRL), with a questionnaire. The results reveal that the SRL-based feedback intervention had a positive impact on EFL student writers’ writing performance as well as their reported use of SRL writing strategies. While the analytic writing scores for the subcategories of organisation, vocabulary and content significantly increased over time for the treatment group, there was little change in language use. ANCOVA analyses suggest significantly positive results for the treatment group in the improvement of SRL writing strategies with goal-oriented monitoring, knowledge rehearsal, feedback handling, and interest enhancement, and the intervention also developed the use of SRL strategies for text processing, idea planning, motivational self-talk, and emotional control.

## Introduction

In EFL/L2 writing, a major issue concerning research on feedback for many years has been the inconsistent findings on the contribution of feedback to the production of L2 texts in both learners’ immediate revisions and their longer-term development as writers. Empirical research on teacher feedback has provided insights on the acquisition of specific linguistic features and the overall quality of writing texts that may derive from feedback processing. While some studies provide evidence of positive effects of feedback, others are equivocal (El Ebyary and Windeatt, 2019). This strand of research has focused on understanding the effect of different types of feedback on textual aspects of student writing and comparing different types of feedback (Bitchener and Storch, 2016). Apart from its potential role in writing performance, feedback is expected to impact the writing process (Hyland and Hyland, 2019; Lee, 2020; Liu and Yu, 2022), an outcome that has received more attention in recent years.

While feedback has often been referred to as “one of the most effective tools to increase learning success” (Hattie and Zierer, 2019, p. 7), its role in motivating students to regulate writing strategies is not clear, especially in the EFL/L2 context. Self-regulation is “a dynamic construct that connects strategic capacity, intent, and learning behaviour” (Dörnyei and Ryan, 2015, p. 169) in the EFL/L2 context. For all self-regulated activities, feedback can serve as a catalyst to moderate learners’ performance processes for the attainment of personal goals (Butler and Winne, 1995; Hattie and Timperley, 2007; Zimmerman and Kitsantas, 2007). It depends on whether the focus of feedback directs at the level of task, process, self-regulation or self as a person (Hattie and Timperley, 2007). Hattie and Timperley (2007) argued that personal feedback did not provide task-related information. While task-level feedback involves how well a task is being completed, feedback can be aimed at the processing of information or learning processes. And the most effective feedback is at the self-regulation level, which engages learners in monitoring, directing, and regulating actions toward the learning goal. In this sense, feedback is not just information on performance, which learners passively receive from an agent. It implies that feedback should focus on promoting learners’ self-regulatory behaviours (Sadler, 1989; Butler and Winne, 1995; Nicol and Macfarlane-Dick, 2006) to achieve “enhanced challenges, more self-regulation over the learning process, greater fluency and automaticity, more strategies and processes to work on the tasks, deeper understanding, and more information about what is and what is not understood” (Wisniewski et al., 2020, p. 2). Following the recent call for more studies on how feedback impacts the writing process, the current study examined whether teacher feedback facilitates, or impedes students’ use of self-regulatory writing strategies in the EFL context.

## Literature review

### Effectiveness of feedback

Prior research has addressed the effectiveness of focused or comprehensive feedback in improving L2 learners’ writing accuracy. Empirical studies on feedback which focus on ideas, rhetorical devices, grammar, lexical use, or mechanics, have reported inconsistent findings (e.g., Sheen, 2007; Bitchener and Knoch, 2010; Shintani et al., 2014; Stefanou and Révész, 2015). Sheen’s (2007) study, for instance, suggested that feedback on a specific linguistic feature improved writing accuracy. Nevertheless, because studies on focused feedback usually targeted only one, or a limited number of, grammatical problems, they may not help teachers in classrooms who encounter multiple language problems in students’ drafts (see Zhang and Cheng, 2021, for a synthesis of research). It is more practical for teachers to target a range of errors in each piece of writing to help students improve written accuracy over time (Lee, 2019).

Although the effectiveness of comprehensive feedback is still controversial, more recent studies (Hartshorn and Evans, 2015; Ferris and Kurzer, 2019; Mak, 2019) have explored how best it can be implemented. Comprehensive feedback was considered ineffective as significant accuracy improvement was found during revision but not in writing a new text (Truscott and Hsu, 2008). However, Van Beuningen et al. (2012) reported that comprehensive feedback led to increased accuracy in an L2 context during both revision and in new writing. Extant literature has also reported the effectiveness of comprehensive corrective feedback on linguistic accuracy (Ashwell, 2000; Ferris and Roberts, 2001) and fluency (Cheng and Zhang, 2021). Despite no conclusive advantage of comprehensive feedback over focused feedback reported, research has investigated how to use comprehensive feedback in L2 writing classrooms. For example, Evans et al. (2010) developed a “dynamic written corrective feedback” (DWCF) approach in an effort to improve writing accuracy “by ensuring that instruction, practice, and feedback are manageable, meaningful, timely, and constant” for both the learner and teacher (Hartshorn and Evans, 2012, p. 30). Given the pedagogical value of comprehensive feedback, more investigation into the implementation of comprehensive feedback is needed.

Another strand of research, comparing the effectiveness of direct feedback, indirect feedback, and metalinguistic feedback, has led to different pedagogical suggestions. In the studies which found direct feedback was more effective than indirect feedback, it was suggested that direct feedback provided explicit information beneficial for students who have difficulty interpreting teachers’ comments (Bitchener and Knoch, 2010; Bonilla López et al., 2018; Zhang and Cheng, 2021). In contrast, some scholars, for instance, Ferris (2006) and Kurzer (2018), have argued for indirect feedback over direct feedback for facilitating EFL students’ writing development over time because indirect feedback “compels students to engage in guided-learning and problem-solving activities” (Lalande, 1982, p. 143). Suzuki et al. (2019), however, confirmed the effectiveness of both direct and indirect feedback on language accuracy but that the effects varied as a function of the type of target structures. Whereas this explains inconsistent findings in the earlier studies and suggests that explicitness may work together with other mediating variables so as to be effective for L2 writing development, it needs further investigation.

Moreover, influenced by process theories in L2 writing, other studies have recommended a balanced coverage of global and local feedback (Ferris, 2003). Previously, feedback research centred on the effect of feedback on the process of writing as well as the product. As such, the focus of teacher feedback included issues of content and organisation (e.g., Cohen and Cavalcanti, 1990; Conrad and Goldstein, 1999). Zamel (1985) warned that extensive attention to grammar errors might divert students’ attention away from developing ideas, or other important concerns, in writing. Ashwell (2000) compared three patterns of feedback: (a) the conventional response (giving feedback on content first and feedback on form in a later draft), (b) the reverse pattern, or (c) one in which form and content feedback were mixed. This study found nonsignificant differences in student gains in accuracy or content scores, which may be a result of a less than explicit understanding of principled feedback patterns by teachers. Lee (2009) identified that teachers in Hong Kong secondary schools provided feedback predominantly on language-related errors but seldom addressed the content, discourse, text structure, and genre aspects of writing. Other studies have revealed similar findings (Montgomery and Baker, 2007; Goldstein and Kohls, 2009). For both writing-to-learn-language and learning-to-write purposes, it is reasonable to consider giving feedback to cover all elements of writing in a balanced way. As a result, Lee (2017) suggested teachers “focus mainly on content and organisation in the first draft and leave language issues to later drafts” (p. 77). More research, however, is needed to understand better how to integrate feedback with the process approach for the overall development of L2 writing.

Although previous studies have recommended an array of valuable principles, suggestions, and strategies to guide teachers when responding to student writing, the controversies surrounding the potential role of feedback for L2 writing development remain. Although L2 writing feedback has recently shifted to being learning-centred (Lee, 2017), the impact of teacher intervention as it influences mediational processes inherent in constructing and revising L2 text needs to be considered (Hedgcock and Lefkowitz, 1994). As such, the study described in this manuscript examines the impact of feedback on the overall development of L2 writers.

### Feedback and self-regulated strategy development in writing

Self-regulated writing is a cyclical process whereby learners use feedback on prior performance to make necessary adjustments to current efforts as personal, behavioural, and environmental factors are constantly changing (Zimmerman, 2000). Feedback may regulate learners’ motivational beliefs, influencing how students feel about themselves, positively or negatively (Dweck, 2000); motivational beliefs, in turn, regulate the effects of feedback messages (Nicol and Macfarlane-Dick, 2006). Viewing feedback in the context of self-regulated learning is to theorize that the most important function of feedback is tutoring, or guiding, the learner to regulate the learning process successfully (Butler and Winne, 1995).

There is a growing interest in concepts of self-regulation in language learning (Jackson and Park, 2020) because of its crucial role in the writing process (Hawe and Dixon, 2014; van der Kleij, 2020). Drawing on sociocultural and social cognitive theories, Teng and Zhang (2016) conceptualized a multidimensional construct of EFL writing strategies for SRL, including cognitive strategies (text processing and knowledge rehearsal), metacognitive strategies (goal-oriented monitoring and idea planning), social behavioural strategies (feedback handling and peer learning), and motivational regulation strategies (motivated self-talk, interest enhancement, and emotional control). The nine sub-strategies in this construct were independent but closely associated (Teng and Huang, 2018; Teng and Zhang, 2021). Sasaki et al. (2018) similarly argued that, as a self-regulation perspective advocates that learning strategies can be affected by cognitive, affective, and environmental factors, it is appropriate for understanding L2 writing strategies. A review of the literature endorses the four domains of self-regulatory development as cognitive processing, motivational beliefs, social behaviour, and metacognitive regulation.

Self-regulated writers are responsive to feedback regarding the effectiveness of their writing skills and the quality of the text they write (Han and Hyland, 2015). This study suggested there are individual differences in setting learning goals, beliefs about the effectiveness of feedback, English writing, and their writing abilities impact their effort and willingness to respond to teachers’ comments. With process-oriented approaches in writing, the role played by feedback is especially important. As Kroll (2001) posited, the process approach engages student writers through multiple drafts wherein they receive external feedback and revise evolving texts. Its cyclical nature resembles that of self-regulatory learning development. The core problem lies in how to implement feedback so as to empower students to be self-regulated writers and “forward the student’s future writing and the development of his or her writing process” (Hyland and Hyland, 2006, p. 83).

There are scant empirical studies investigating the link between feedback and self-regulatory learning in relation to EFL writing. However, Lam (2015) validated the argument that feedback in portfolio assessment could harness formative benefits to enhance SRL. These studies were carried out in a Hong Kong secondary school and a Hong Kong university, where the process-oriented approach was adopted for writing. Based on the results, Lam suggested that feedback on self-regulation in portfolio assessment settings might develop students’ self-regulatory capacity and help in closing the learning gap. Adopting the framework in Pintrich’s (2000) model of self-regulation, Mak and Wong (2018) also conducted a 1-year multiple case study, which explored how teachers in two elementary schools in Hong Kong implemented portfolio assessment to develop students’ self-regulation capacity. This study confirmed that portfolio assessment was effective in empowering students to be self-regulated writers as it developed student writers’ agency, goal-orientation, openness to feedback, and enhanced their self-evaluation and self-reflection capacity. Vasu et al. (2020), in comparing the effectiveness of self-assessment and indirect teacher feedback on students’ SRL in L2 writing, revealed that both helped to develop self-regulated learners. Lam (2015), however, concluded that debates over how teachers can make productive use of feedback to promote SRL remain ‘unfinished business’ in L2 writing research and that further examination of the role of feedback in encouraging learners to become self-regulated writers is needed.

### Self-regulated learning-based feedback loop

From a social cognitive perspective, self-regulation is viewed as an interaction of three cyclical phases: forethought, performance, and a self-reflection process (Zimmerman and Campillo, 2003). During these phases, feedback, whether internally or externally generated, is critical to the self-regulatory process as it provides information as to the quality and effectiveness of the learning strategies and processes employed to meet their goals (Butler and Winne, 1995). To ensure students become self-regulated writers, teachers need to provide feedback to support self-regulation.

To ensure feedback for learning is effective, the teacher needs to cue students’ attention to the learning task, task processing strategies and self-regulation strategies (Hattie and Gan, 2011). Hattie and Timperley (2007) developed a feedback model which relates to three key questions and four major dimensions of learning and learners. The three questions are “Where am I going?” “How am I going?” and “Where to next?” The effectiveness of feedback can differ, depending on the answers to these questions, that is, whether they are operated at the level of task performance, the level of process, the regulatory or metacognitive level, and/or the self or personal level. Hattie and Timperley (2007) claimed effectiveness was determined according to whether the feedback is about the task (FT), the processing of the task (FP), self-regulation (FR), and/or the self as a person (FS). They also argued that the most effective feedback moved students from task to processing and then from processing to regulation. However, little research has investigated the effectiveness of process-SR feedback on writing development in the EFL/L2 context.

In this study, a three-stage scaffolding feedback loop, framed within the SRL theory and feedback model of Hattie and Timperley (2007), was designed to foster self-regulated writers. During the initial forethought phase, learners are guided to think about the question of “where am I going” by understanding writing criteria and setting goals. During the next performance phase, “feeding back” conveys information about students’ strengths and weakness, progress, and about how to proceed so that students can use the feedback to monitor their progress toward goals. Based on suggestions for quality feedback, feedback at the level of process and self-regulation was offered on the content and organisation of the first drafts, and the vocabulary and language use of the second drafts. During the self-reflection phase, the students self-assess their writing process and track their learning in the forms of reflective journals, goal checklists or reflection sheets.

To examine the effectiveness of SRL-based feedback on the development of self-regulated writing strategies in the EFL context, this study employed a quasi-experimental design in which the following two research questions were addressed:

(1) Does the SRL-based teacher feedback enhance students’ writing performance as seen in the various components such as content, organisation, vocabulary, language use and overall writing scores?
(2) Does the SRL-based teacher feedback enhance students’ self-regulated writing strategies?

## Methodology

### Context and participants

The study was undertaken at a medium-ranking university in the south-eastern part of China. Students admitted to the English studies programme of this university take courses in language skills (phonetics, grammar, listening, speaking, reading, writing, and translation), English language and literature study (linguistics, English cultures, and English culture) and relevant knowledge in Business English.

Seventy participants from two parallel intact classes of English major sophomores were recruited on a voluntary basis. The two classes were randomly assigned to a treatment group or control group. The two groups were comparable in educational background, years of English learning, age, and gender (see Table 1). Their average global writing band score in the Academic International English Language Testing System (IELTS) was 5.5. All these students were enrolled in the same two-credit English writing course, which met once a week for 90 mins over 16 weeks. The course, taught in English, develops students’ understanding, knowledge, and skills in narrative, expository and argumentative writing in English. At the time of the study, genre and function writing approaches were being taught. Both classes were taught by an experienced ESL teacher who spoke Mandarin Chinese, had a master’s degree in applied linguistics, and with over 10 years of teaching experience.

**TABLE 1:** Demographic information of the treatment and control groups.

Groups	Number of students		Mean age	Years of EFL studies
	Female	Male		
Treatment group	32 (91.4%)	3 (8.6%)	19.29 (*SD* = .83)	9.71 (*SD* = 1.47)
Control group	32 (91.4%)	3 (8.6%)	19.34 (*SD* = .64)	9.71 (*SD* = 1.38)

### Data collection

This quasi-experimental study was conducted over 15 weeks, consisting of three testing sessions and three rounds of treatment sessions. The procedures of how the testing and treatment sessions were conducted are presented below (see Table 2).

**TABLE 2:** Procedures of data collection.

Week	Treatment group	Control group
1	Pretest + questionnaire	
2	Draft 1 (Text 1)	Text 1
3	Writing draft 2 (Text 1)	
4	Writing final draft (Text 1)	
5	Draft 1 (Text 2)	Text 2
6	Writing draft 2 (Text 2)	
7	Writing final draft (Text 2)	
8	Draft 1 (Text 3)	Text 3
9	Writing draft 2 (Text 3)	
10	Writing final draft (Text 3)	
11	Posttest + questionnaire	
15	Delayed posttest	

#### Writing tests

A writing test was included to measure whether the intervention affected students’ writing performance over time. All writing tests were chosen from Writing Task Two of IELTS because: (1) it is an international high-stakes test that provides reliable evidence of a person’s English proficiency (Green, 2007), and (2) the requirements of Task 2 are similar to those of the students’ writing course. The course objectives require students to draft an argumentative essay of 200–300 words by the end of the term. The second writing task of IELTS, likewise, asks the examinees “to provide general factual information, outline and/or present a solution, justify an opinion, and evaluate ideas and evidence” (UCLES, 2002). To guarantee the difficulty of writing topics, two experienced writing instructors were invited to evaluate levels of task difficulty and judge whether the second-year undergraduates would be interested in, and knowledgeable about, the ten potential topics; and they then chose three topics of similar difficulty.

Pre-intervention writing tests were given in the first week of the semester to all participants. Post-test was administered after the treatment, while the delayed post-test was carried out 1 month after the intervention to investigate the sustainability of the effects.

#### Questionnaire

The current study adopted the Writing Strategies for the Self-regulated Learning Questionnaire (WSSRLQ) developed by Teng and Zhang (2016) to measure the students’ reported use of writing strategies for SRL. This self-report questionnaire was designed from the perspective of self-regulated writing strategies (Teng and Huang, 2018), measuring four dimensions of self-regulation: cognition, metacognition, social behaviour, and motivational regulation. A 7-point Likert scale ranging from 1 (not at all true of me) to 7 (very true of me) was used for all the items. Nine items ask students to rate their use of cognitive strategies for text processing and knowledge rehearsal; nine items evaluate how well students use metacognitive writing strategies for idea planning and goal-oriented monitoring and evaluating; seven items investigate social-behavioural strategies in peer learning and feedback handling; and fifteen items ask how well students regulate motivation by applying interest enhancement, motivational self-talk, and emotional control. The reliability and validity of the questionnaire in EFL writing contexts were calculated with satisfactory results (Teng and Zhang, 2016). The validated research setting was at the tertiary education level, which was very similar to our research context. In our sample, the internal consistency reliability coefficients (Cronbach’s alpha) for each of the nine sub-strategies ranged from 0.85 to 0.93, suggesting robust reliability.

#### Treatment

Participants in the treatment group were involved in the SRL-based feedback loop (Table 3). Prior to the intervention, they received workshop training to familiarise themselves with the multiple-draft writing practice and feedback activities. Before each writing task, students in the treatment group were required to set up their writing goals by referring to writing criteria. On receiving the teacher’s feedback on the first and second drafts, they revised accordingly and kept an error log in which they recorded errors, changes, achievements, and problems in both drafts. By the end of each writing task, students in the treatment group completed a prompted reflective journal documenting changes between drafts and attributions. Only the treatment group experienced the SRL-based feedback loop for each of the three writing tasks.

**TABLE 3:** Feedback loop for the treatment and control groups.

Draft	Treatment group	Control group
1	• Setting writing goals • Writing the first draft • Receiving feedback on content and organisation • Keeping an error log	• Writing a single draft • Receiving feedback on content, organisation, vocabulary and language use • Receiving an analytic and holistic evaluation
2	• Revising the first draft • Receiving feedback on vocabulary and language use • Keeping an error log	___
3	• Revising the second draft • Receiving an analytic and holistic evaluation • Writing a reflective journal	___

As for the feedback on drafts, students in the treatment group received feedback at the process-SR level. Feedback at the process level was linked to the processes a student would need to perform or understand to complete a task, whereas feedback at the self-regulation level was directed toward the student’s self-management and self-evaluation of the task being completed. Because feedback at these two levels is closely linked, it was often difficult to distinguish between them in written feedback (Hattie and Timperley, 2007). To address this issue, the present study adopted la Fata Almendral’s (2014) feedback model and combined these two levels (Appendix A).

The control group completed a single draft for each writing task and received conventional feedback once at the task level. Task level feedback, received by the control group, was mainly direct corrections that the teacher offered on content, organisation, vocabulary, and language (Appendix A).

### Data analysis

The assumptions of the statistical tests such as the normality of distribution, missing values, outliers, and Levene’s test for homogeneity of variance were checked. Before proceeding with statistical analysis of the impact of the intervention, no significant differences in targeted variables of writing performance and use of SRL writing strategies were identified (see section “Comparability of groups at the onset of the study”).

Writing performance was scored based on UCLES (2002) composite profile, a well-established writing rubric that comprises five subscales: content, organisation, vocabulary, language use, and mechanics: The scale for content is 30%, organisation 20%, language use 25%, vocabulary 20%, and mechanics 5%. It was chosen to capture variation across the different subcomponents that made up the totality of writing skills over time, and because it was reported to produce more reliable and valid scores than other rubrics (Polio, 2013).

Using this scoring rubric, two expert raters evaluated the essays. Both raters held a master’s degree in applied linguistics and were experienced ESL writing teachers. To establish a conservative estimate of interrater reliability, intraclass correlation coefficient (ICC) was employed (Stemler and Tsai, 2008). A high degree of reliability was found between the two raters’ measurements. The intra-rater coefficient for Rater One was *r* = 0.93, *p* < 0.001 and for Rater Two it was *r* = 0.86, *p* < 0.001. The inter-rater reliability between the raters to assess the grades was reasonably high at 0.86, *p* < 0.001. Consistency estimates showed that the two raters reached acceptable inter-rater reliability (Pearson’s *r*: total score = 0.87; content = 0.78; organisation = 0.81; vocabulary = 0.80; language use = 0.76; mechanics = 0.80, i.e., the scales in the rubric considered interval scales) (Brown, 2014). This study used the average scores of the two raters to examine changes in overall writing performance, as well as in content, organisation, language, and vocabulary, respectively.

For both research questions, a series of *t*-tests were employed to examine the overall writing score and changes in use of SRL writing strategies within groups. A series of one-way repeated measures analyses of covariance (ANCOVA) was then performed to determine if there was a statistically significant difference among the post-intervention scores over the semester by controlling for pre-intervention scores. Analytic writing scores are not normally distributed; therefore, non-parametric tests including Wilcoxon signed rank tests and Mann-Whitney *U* tests were conducted.

## Results

### Comparability of groups at the onset of the study

Before proceeding with the statistical analysis of the impact of the intervention, the current study verified there were no significant differences in targeted variables of writing performance and SRL writing strategies.

An independent samples *t*-test was conducted to compare holistic and analytic writing scores of the pre-test between the control and treatment group. Table 4 depicts the means (*M*) and standard deviations (*SD*) of the pre-test scores for both the control (*M* = 65.90, *SD* = 7.87) and treatment groups (*M* = 67.50, *SD* = 8.58). Independent samples *t*-tests showed no statistically significant group differences in the pre-test total scores between groups, *t* (68) = 0.81, *p* = 0.42. There was no significant difference in the analytic scores of content, vocabulary, language use and mechanics, except organisation, *t* (68) = –2.08, *p* = 0.04. These findings confirmed that the two groups were comparable in terms of writing performance at the onset of the study.

**TABLE 4:** Descriptive statistics and independent samples *t*-tests of writing test scores for the control and treatment groups in the pre-test.

Writing test scores	Group	*N*	*M*	*SD*	*t*	*p*	95% *CI*	
							*LL*	*UL*
Content	TRE	35	18.54	3.25	–1.28	0.20	–2.59	0.56
	CON	35	17.53	3.37				
Organisation	TRE	35	13.01	1.66	–2.08	0.04*	–1.65	–0.04
	CON	35	12.17	1.73				
Vocabulary	TRE	35	14.24	1.48	0.30	0.77	–0.57	0.77
	CON	35	14.34	1.31				
Language use	TRE	35	18.04	2.32	0.28	0.78	–0.88	1.17
	CON	35	18.19	1.95				
Mechanics	TRE	35	3.76	.52	–0.97	0.34	–0.35	0.12
	CON	35	3.64	.46				
Total scores	TRE	35	65.90	8.58	–0.81	0.42	–5.53	2.33
	CON	35	67.50	7.87				

*p < 0.05; **p < 0.001. A weighted sum was used to represent the relative importance of five aspects of writing performance in the total score: content (30%, 13–30 points), organisation (20%, 7–20 points), language (25%, 5–25 points), vocabulary (20%, 7–20 points) and mechanics (5%, 2–5 points). CI, confidence interval; LL, lower limit; UL, upper limit.

The differences in their use of SRL writing strategies were also calculated through independent *t*-tests. As shown in Table 5, no significant differences were found in the use of all the nine SRL writing strategies between the two groups at the beginning of the study.

**TABLE 5:** Descriptive statistics and independent samples *t*-tests of self-regulated learning (SRL) strategies scores for the control and treatment groups in the pre-test.

SRL strategies		Group	*N*	*M*	*SD*	*t*	*p*	95% *CI*	
								*LL*	*UL*
Cognitive	Text processing	TRE	35	4.93	1.02	–1.17	0.25	–0.76	0.20
		CON	35	4.65	0.99				
	Knowledge rehearsal	TRE	35	4.83	0.86	0.37	0.71	–0.34	0.49
		CON	35	4.91	0.87				
Metacognitive	Idea planning	TRE	35	4.67	1.00	0.99	0.33	–0.22	0.66
		CON	35	4.89	0.84				
	Goal-oriented monitoring	TRE	35	4.52	0.984	–0.19	0.85	–0.48	0.34
		CON	35	4.48	0.86				
Social behavioural	Peer learning	TRE	35	3.70	1.38	1.18	0.24	–0.27	1.05
		CON	35	4.09	1.38				
	Feedback handling	TRE	35	4.61	0.85	–0.13	0.90	–0.48	0.42
		CON	35	4.58	1.01				
Motivational regulation	Interest enhancement	TRE	35	4.75	0.99	–0.20	0.84	–0.58	0.48
		CON	35	4.70	1.21				
	Motivational self-talk	TRE	35	5.22	0.91	0.50	0.62	–0.33	0.55
		CON	35	5.33	0.92				
	Emotional control	CON	35	4.94	0.95	–1.03	0.31	–0.76	0.24
		TRE	35	4.70	1.13				

This is a 7-point Likert scale; 1 = not at all true of me; 2 = not true of me; 3 = slightly not true of me; 4 = neutral; 5 = slightly true of me; 6 = true of me; 7 = very true of me. CI, confidence interval; LL, lower limit; UL, upper limit.

### Changes in writing test scores within and between groups

The effect of SRL-based feedback on writing performance was measured by holistic writing scores and analytic writing scores of subcategories: content, organisation, vocabulary, and language use.

#### Holistic writing test scores

As shown in Table 6, *t*-tests indicate that the treatment group manifested significant gains in the overall writing test scores in the post-test (*M* = 75.97, *SD* = 5.51), *t* (34) = 5.98, *p* < 0.001 and the delayed post-test (*M* = 75.77, *SD* = 5.17), *t* (34) = –5.83, *p* < 0.001. The magnitude of gains was large in the post-test (Cohen’s *d* = 0.85) and in the delayed post-test (Cohen’s *d* = 0.84) (Cohen, 1988).

**TABLE 6:** Paired samples *t*-tests of holistic writing scores in the pre-, post-, and delayed post-tests for the treatment and control groups.

Group	*N*	Pre-test		Post-test		Pre-test vs. Post-test			Delayed Post-test		Pre-test vs. Delayed post-test		
		*M*	SD	*M*	SD	*t*	*p*	Cohen’s *d*	*M*	SD	*t*	*p*	Cohen’s *d*
TRE	35	67.50	8.58	75.97	5.51	–5.98	<0.001**	0.85	75.77	5.17	–5.83	<0.001**	0.84
CON	35	65.90	7.87	72.74	5.10	–4.60	<0.001**	0.78	71.80	4.97	–4.25	<0.001**	0.72

*p < 0.05; **p < 0.001. TRE, treatment group; CON, control group; M, mean; SD, standard deviation.

Equally, students from the control group improved their overall writing scores significantly in the post-test (*M* = 72.74, *SD* = 5.10); *t* (35) = –4.60, *p* < 0.001 and in the delayed post-test (*M* = 71.80, *SD* = 4.97); *t* (35) = –4.25, *p* < 0.001. The values of Cohen’s *d* = 0.78 in the post-test and Cohen’s *d* = 0.72 in the delayed post-test suggest a medium-size effect.

Results of ANCOVA showed significant differences in overall writing gains between the two groups in the post-test [*F* (1, 68) = 5.71, *p* = 0.02, partial *η^2^* = 0.08] and in the delayed post-test [*F* (1, 68) = 9.87, *p* = 0.002, partial *η^2^* = 0.13]. The covariate was also significant: *F* (1, 68) = 4.56, *p* = 0.04, partial *η^2^* = 0.06 for the post-test and *F* (1, 68) = 6.38, *p* = 0.01, partial *η^2^* = 0.09 for the delayed post-test, indicating that students’ pre-writing scores had a significant effect on their writing gains at the post- and delayed post-tests after the intervention. ANCOVA results reveal that the treatment group performed significantly better than the control group in the post-test with a medium-size effect. These findings suggest that the impact of SRL-based feedback practice on students’ writing test performance is positive, and its effect is sustained for at least 1 month. In addition, students’ pre-test writing ability appeared to influence their writing performance in the post and the delayed post-tests.

#### Analytic writing test scores

The results of Wilcoxon signed rank tests summarised in Table 7 reveal that the treatment group showed a significant positive change following participation in the SRL-based feedback intervention with a large to medium-size effect in content, *Md* = 22.50, *z* = –4.37, *p* < 0.001, *r* = 0.52; in organisation, *Md* = 15.00, *z* = –4.38, *p* < 0.001, *r* = 0.52; in vocabulary, *Md* = 15.50, *z* = –3.69, *p* < 0.001, *r* = 0.44; in language, *Md* = 20.00, *z* = –3.68, *p* < 0.001, *r* = 0.44. The gains of the four categories in the treatment group were all retained 1 month after the intervention with medium to large-sized effects : *Md* = 22.00, *z* = –4.51, *p* < 0.001, *r* = 0.54; *Md* = 15.00, *z* = –4.60, *p* < 0.001, *r* = 0.55; *Md* = 15.50, *z* = –4.02, *p* < 0.001, *r* = 0.48; *Md* = 19.00, *z* = –3.06, *p* < 0.001, *r* = 0.36.

**TABLE 7:** Descriptive statistics and results of Wilcoxon signed rank tests of analytic writing scores for the treatment and control groups in the pre-, post-, and delayed post-tests.

Subcategories	Group	*N*	Pre-test	Post-test	Pre-test vs. Post-test		Delayed post-test	Pre-test vs. Delayed post-test	
			*Md*	*Md*	*Z*	*p*	*Md*	*Z*	*p*
Content	TRE	35	18.00	22.50	–4.37	0.000**	22.00	–4.51	0.000**
	CON	35	17.50	21.00	–4.15	0.000**	21.00	–3.95	0.000**
Organisation	TRE	35	13.00	15.00	–4.38	0.000**	15.00	–4.60	0.000**
	CON	35	12.00	14.50	–3.82	0.000**	14.00	–4.29	0.000**
Vocabulary	TRE	35	14.50	15.50	–3.69	0.000**	15.50	–4.02	0.000**
	CON	35	14.00	15.00	–2.15	0.032*	14.50	–1.68	0.094
Language use	TRE	35	18.50	20.00	–3.68	0.000**	19.00	–3.06	0.002*
	CON	35	18.00	19.50	–2.71	0.007*	14.50	–1.03	0.303

*p < 0.05; **p < 0.001. TRE, treatment group; CON, control group.

The control group, similarly, indicated a significant increase in all of four subcategories by the end of the writing course: content, *Md* = 21.00, *z* = –4.15, *p* < 0.001, with a large-size effect (*r* = 0.50); organisation, *Md* = 14.50, *z* = –3.82, *p* < 0.001, with a medium-size effect (*r* = 0.46); vocabulary, *Md* = 15.00, *z* = –2.15, *p* = 0.032, with a small-size effect (*r* = 0.26); language use, *Md* = 19.50, *z* = –2.71, *p* = 0.007, with a medium-size effect (*r* = 0.32). For content and organisation there was evidence of significant improvement at the delayed post-test, in content, *z* = –3.95, *p* < 0.001, with a medium effect size (*r* = 0.47); in organisation, *z* = –4.29, *p* < 0.001, with a large effect size (*r* = 0.51).

A series of Mann-Whitney *U* tests were performed to examine whether there was a significant difference between the control and treatment groups in their post-intervention and delayed post-intervention analytic writing scores in four subcategories. As shown in Table 8, the treatment group significantly outperformed the control group in three subcategories: content (*U* = 403.00, *z* = –2.47, *p* = 0.013, *r* = 0.42), organisation (*U* = 346.50, *z* = –3.15, *p* = 0.002, *r* = 0.53) and vocabulary (*U* = 422.50, *z* = –2.28, *p* = 0.023, *r* = 0.39) in the post-test, with a medium to large-size effect. At the delayed post-test the gains were significant for content (*U* = 305.00, *z* = –3.63, *p* < 0.001, *r* = 0.61), organisation (*U* = 372.00, *z* = –2.86, *p* = 0.004, *r* = 0.48) and vocabulary (*U* = 430.00, *z* = –2.18, *p* = 0.03, *r* = 0.36). No significant difference was found between the two groups in the analytic writing scores for language use at the delayed post-test.

**TABLE 8:** Descriptive statistics and results of Mann-Whitney *U* tests of analytic writing scores for the treatment and control groups in the post- and delayed post-tests.

Subcategories	Group	*N*	Post-test				Delayed post-test			
			***Md***	***U***	***z***	***p***	***Md***	***U***	***Z***	***p***
Content	TRE	35	22.50	403.00	–2.47	0.013*	22.00	305.00	–3.63	0.000**
	CON	35	21.00				21.00			
Organisation	CON	35	14.50	346.50	–3.15	0.002*	14.00	372.00	–2.86	0.004*
	TRE	35	15.00				15.00			
Vocabulary	CON	35	15.00	422.50	–2.28	0.023*	14.50	430.00	–2.18	0.03*
	TRE	35	15.50				15.50			
Language use	CON	35	19.50	493.50	1.407	0.16	19.00	483.50	–1.53	0.13
	TRE	35	20.00				19.00			

*p < 0.05; **p < 0.001. TRE, treatment group; CON, control group

### Changes in self-regulated learning writing strategies within and between groups

#### Cognitive strategies

Descriptive statistics in Table 9 showed that students from the treatment group increased their use of both text processing (*M* = 4.93, *SD* = 1.02 for the pre-test; *M* = 5.22, *SD* = 0.74 for the post-test) and knowledge rehearsal strategies (*M* = 4.83, *SD* = 0.86 for the pre-test; *M* = 5.23, *SD* = 0.84 for the post-test) over time. The magnitude of the changes in the means of text processing strategies [*t* (34) = –2.16, *p* = 0.04, Cohen’s *d* = 0.33] and knowledge rehearsal strategies [*t* (34) = –3.00, *p* = 0.01, Cohen’s *d* = 0.47] was small.

**TABLE 9:** Descriptive statistics and results of paired-samples *t*-tests of SRL strategies for the treatment and control groups in the pre- and post-tests.

SRL strategies		Group	*N*	Pre-test		Post-test		*t*-test	
				*M*	*SD*	*M*	*SD*	*t*	*P*
Cognitive	Text processing	CON	35	4.65	0.99	4.88	0.16	–1.17	0.25
		TRE	35	4.93	1.02	5.22	0.74	–2.16	**0.04** *
	Knowledge rehearsal	CON	35	4.91	0.87	4.81	1.08	0.68	0.50
		TRE	35	4.83	0.86	5.23	0.84	–3.00	**0.01** *
Metacognitive	Idea planning	CON	35	4.89	0.84	5.17	0.82	–1.47	0.15
		TRE	35	4.67	1.00	5.11	0.75	–2.87	**0.01** *
	Goal-oriented monitoring	CON	35	4.48	0.86	4.66	0.91	–1.07	0.29
		TRE	35	4.52	0.98	5.06	0.74	–3.27	**0.002** *
Social behavioural	Peer learning	CON	35	4.09	1.38	3.99	1.19	0.30	0.77
		TRE	35	3.70	1.38	3.81	1.04	–0.47	0.64
	Feedback handling	CON	35	4.58	1.01	4.73	1.07	–0.76	0.45
		TRE	35	4.61	0.85	5.49	0.74	–7.18	**<0.001** **
Motivational regulation	Interest enhancement	CON	35	4.70	1.21	4.71	1.08	–0.04	0.97
		TRE	35	4.75	0.99	5.19	0.84	–2.64	**0.01** *
	Motivational self-talk	CON	35	5.33	0.92	5.56	0.98	–1.34	0.19
		TRE	35	5.22	0.91	5.74	0.68	–4.02	**<0.001** **
	Emotional control	CON	35	4.70	1.13	5.01	0.89	–1.60	0.12
		TRE	35	4.95	0.95	5.31	0.64	–2.18	**0.04** *

*p < 0.05; **p < 0.001. TRE, treatment group; CON, control group.

For the control group, the mean scores of text processing strategy in the post-test (*M* = 4.88, *SD* = 0.16) were higher than that of the pre-test (*M* = 4.65, *SD* = 0.99), but this difference was not significant [*t* (34) = –1.17, *p* = 0.25]. The mean scores of knowledge rehearsal strategy declined slightly from pre-test (*M* = 4.91, *SD* = 0.87) to post-test (*M* = 4.81, *SD* = 1.08).

Through subsequent ANCOVA analysis, significant differences, *F* (1, 68) = 6.61, *p* = 0.01, between the mean scores of the control and treatment groups in the reported knowledge rehearsal strategy were evident. The eta-squared effect size (*η^2^* = 0.09) was a medium effect by Cohen’s benchmarks. No significant difference was found in the use of text processing between the two groups after the intervention. However, the covariate of the pre-test was significant with *F* (1, 68) = 13.89, *p* < 0.001, partial η^2^ = 0.17 for text processing and *F* (1, 68) = 36.68, *p* < 0.001, partial η^2^ = 0.35 for knowledge rehearsal. This suggests that students’ prior cognitive strategies influenced the use of text processing strategies with a moderate effect size and knowledge rehearsal with a large effect size.

#### Metacognitive strategies

When comparing the use of metacognitive strategies, students in the treatment group significantly increased their use of idea planning *t* (34) = 2.87, *p* = 0.01 and goal-oriented monitoring *t* (34) = 0.55, *p* = 0.002 between the pre-test and the post-test. The magnitude of increases was moderate for idea planning (Cohen’s *d* = 0.50) and large for goal-oriented monitoring (Cohen’s *d* = 0.62). However, no significant change in the use of metacognitive strategies was found in the control group. To compare the use of idea planning and goal-oriented monitoring with the treatment condition, ANCOVAs were conducted. The results revealed a small effect for the use of goal-oriented monitoring, *F*(1,68) = 4.20, *p* = 0.04, partial η^2^ = 0.06 between the control and treatment groups, with no significant difference between the two groups in regard to idea planning, *F*(1,68) = 0.006, *p* = 0.94. The covariate, however, was significantly related to idea planning *F*(1,68) = 5.80, *p* = 0.02, partial η^2^ = 0.08 and goal-oriented monitoring *F*(1,68) = 9.31, *p* = 0.003, partial η^2^ = 0.12. This meant that students’ pre-existing level of using these two metacognitive strategies had a small effect.

#### Social behavioural strategies

With social behavioural strategies, the descriptive statistics revealed an increase in the mean scores of peer learning of the treatment group (*M* = 3.70, *SD* = 1.38; *M* = 3.81, *SD* = 1.04), however, it was not significant, *t* (34) = –0.47, *p* = 0.64. The use of feedback handling showed a substantial growth, increasing from *M* = 4.61 (*SD* = 0.85) in the pre-test to *M* = 5.49 (*SD* = 0.74) in the post-test. Paired samples *t*-tests also revealed significant changes in using the feedback handling strategy, *t* (34) = 7.18, *p* < 0.001, with a strong effect size (Cohen’s *d* = 1.10) in the treatment group. However, with the control group, there was no significant change in peer learning [*t* (34) = 0.30, *p* = 0.77] nor feedback handling [*t* (34) = –0.76, *p* = 0.45] by the end of the writing course.

The ANCOVA indicated that, even though the treatment group rated peer learning higher than the control group, the differences were not significant. The scores for feedback handling in both the control and treatment groups increased over time. Results of ANCOVA demonstrated that there was a significant group difference, with a medium effect, for feedback handling, *F* (1, 68) = 13.69, *p* < 0.001, partial η^2^ = 0.17. The *F* statistic for the pre-test was significant with *F* (1, 68) = 15.10, *p* < 0.001, partial η^2^ = 0.18 for feedback handling.

#### Motivational regulation strategies

In the treatment group, there was an increase in scores for the three motivational regulation strategies of interest enhancement, motivational self-talk, and emotional control at the post-test. Based on paired samples *t*-tests, the increase between the pre-test and the post-test in each category was statistically significant: interest enhancement *t* (34) = 2.64, *p* = 0.01; motivational self-talk *t* (34) = 4.02, *p* < 0.001, and emotional control *t* (34) = 2.18, *p* = 0.04. The magnitude of the differences was small for interest enhancement (Cohen’s *d* = 0.48) and emotional control (Cohen’s *d* = 0.44), and medium for motivational self-talk (Cohen’s *d* = 0.65).

For the students in the control group, interest enhancement was unchanged although they increased in the use of motivational self-talk (*M* = 5.22, *SD* = 0.91 for the pre-test; *M* = 5.74, *SD* = 0.68 for the post-test) and emotional control (*M* = 4.95, *SD* = 0.95 for the pre-test; *M* = 5.31, *SD* = 0.64 for the post-test). The changes were not significant in the paired samples *t*-tests.

The ANCOVAs demonstrated that there was a significant group difference only in interest enhancement, *F* (1, 68) = 4.68, *p* = 0.03, with a small effect size (partial *η^2^* = 0.07). No significant difference was found in motivational self-talk and emotional control between the two groups at the post-test. The covariate of pre-test scores had a significant influence on interest enhancement *F* (1, 68) = 11.07, *p* = 0.001, partial *η^2^* = 0.14; motivational self-talk *F* (1, 68) = 21.20, *p* < 0.001, partial η^2^ = 0.24; and emotional control *F* (1, 68) = 9.13, *p* = 0.004, partial η^2^ = 0.12 with a medium effect size.

## Discussion

The purpose of this study was to explore the effects of an SRL-based feedback practice on EFL learners’ writing performance and the use of SRL writing strategies in a tertiary context. SRL-based feedback activities were implemented and process-SR feedback was provided for the intervention group, whereas the control group completed the same writing tasks with conventional task-level feedback.

The first research question sought to determine whether the SRL-based feedback practice would enhance students’ EFL writing as measured by holistic scores and analytic scores in the four subcategories (content, organisation, vocabulary, and language use). Although students in the control group indicated a significant increase in overall writing performance and all four subcategories, the SRL-based feedback practice facilitated the treatment group to significantly outperform the other in overall writing scores, and the content, organisation, and vocabulary of their text over time.

The feedback given to students in the treatment group, mainly at the process and self-regulation level, appeared to contribute to overall writing performance. Hattie and Clarke (2019) have argued that the most valuable feedback provides “where to next” or “how to improve this work” information (p. 2). Likewise, Dixon and Hawe (2017) claimed that for feedback to be effective, it needs to explain misconceptions and suggest actions necessary for improving future work. Participants in the treatment group in this study were given process-SR feedback to support their process of reviewing writing knowledge, using cognitive strategies, and monitoring and regulating learning through planning and adapting learning strategies when necessary. There is evidence to show that effective feedback within a process writing approach can lead to improved writing performance. For example, Lam (2013), positing that a feedback-rich environment could lead to improved writing performance, implemented two writing portfolio assessment systems to facilitate process writing and peer review. In another study, Mak, 2019 reported students perceived potential for improving their writing performance after participating in an integrated three-stage feedback practice into a holistic feedback process. The effect of the SRL-based feedback practice of integrating feedback into the process writing approach in the present study, is consistent with these two studies. The treatment group students, in completing the steps involved in drafting and redrafting a piece of written work, engaged in a process through which they produced, reflected on and revised successive drafts of a text.

The progress the treatment group made in the content and organisation scores might be due to the focused approach to feedback; that is, providing feedback on content and organisation for the first draft and on vocabulary and language use for the second draft. Previous research (Hartshorn and Evans, 2015; Cheng and Zhang, 2021) demonstrated little improvement in content and organisation. However, in these studies comprehensive feedback was offered on content, organisation and writing accuracy, while students tended to prioritize linguistic errors in revision (Chen and Zhang, 2019). In the present study, students were given equal opportunity to correct errors in content, organisation, vocabulary, and language use. Ashwell (2000) similarly found no significant differences between a treatment and comparison group in content scores. Three patterns of feedback were noted: the conventional response, that is, (a) giving feedback on content first and feedback on form later: (b) the reverse pattern: or (c) one in which form and content feedback were mixed. A point of difference for students in the present study is that they were guided to understand why the focus of feedback was different for each draft. Furthermore, the requirement for them to record their writing performance, and reflect on their writing process and work, after revising global issues of the texts, may have directed their attention to content and organisation issues in their writing.

There was also a significant improvement in the treatment group’s vocabulary, thus providing further evidence for the efficiency of indirect comprehensive feedback on lexical errors. Feedback at the process level may be better suited for lexical errors than corrections at the task level as the former may activate in-depth processing of lexical features and foster long-term acquisition (Ferris and Roberts, 2001; Van Beuningen et al., 2012). More recent research (Cheng and Zhang, 2021) revealed that a teacher’s corrections seemed to be ineffective in improving lexical complexity. In the present study, process-SR feedback was used to encourage the students themselves to review their linguistic knowledge, which, as Buckingham and Aktuğ-Ekinci (2017) posited, may have longer-term learning benefits for lexical use.

Both treatment and control groups showed an increase in language use and grammar, with the treatment group not significantly outperforming the control group. The finding suggests that an indirect approach to feedback in the treatment group may not be more efficient in improving language use and grammar than a direct feedback approach where corrections are provided, as in the control group. It was expected that indirect feedback could guide the learners to refer to their existing knowledge and consequently self-correct errors (Ferris, 2006; Kurzer, 2018). This strategy, as argued by Bitchener and Knoch (2010), may be more effective in the long term. It should be noted that while indirect feedback at the process and self-regulation level created opportunities for participants in the treatment group to engage in problem-solving activities, these opportunities can be cognitively demanding for some students with limited language proficiency when trying to interpret the teacher’s feedback (Bonilla López et al., 2018; Zhang and Cheng, 2021).

There was a statistically significant increase in using SRL strategies of knowledge rehearsal, goal-oriented monitoring, feedback handling, and interest enhancement for the treatment group following the intervention. There were no statistically significant changes in using the other five SRL strategies of text processing, idea planning, peer learning, motivational self-talk, and emotional control. The significant increase in knowledge rehearsal can be explained by the participants’ long-entrenched practice of the learning strategy of memorizing. As a result of EFL instructors encouraging students to focus on vocabulary in writing, most EFL/L2 writers consider a robust command of vocabulary as the main criterion for an excellent essay (Zamel, 1985; Yasuda, 2015). It is not surprising that students in the present study initially favoured a memorization strategy to build vocabulary as a lexical resource during the composing episode.

The SRL-based feedback intervention seemed to greatly increase students’ use of the metacognitive strategy of goal-oriented learning. In this study, students were requested to keep an error log to self-reflect on errors in their current writing assignment and to set goals for a new assignment. Keeping an error log encourages students to commit to goals to further their learning (Wiliam, 2011; Mak, 2019). Zimmerman (2011) reviewed the evidence supporting the conscious setting of appropriate goals to optimize learning which included studies describing goals recorded in an error log which were sufficiently specific to be measured with criteria-based feedback. Carver and Scheier (1991) posited that goal-setting was a conscious process in which students decide what self-information they need in order to develop self-regulation. It is possible that recording goal setting in an error log encourages students to consider how they plan to act on feedback comments and set goals. In the present study, the reflective journals also appeared to activate students’ awareness of the benefits of self-reflection on writing outcomes and acquiring new writing knowledge.

Students from the treatment group in the current study made significant gains in handling feedback, with a large effect size, in comparison to the effect sizes for the other self-regulated strategies. Feedback handling involves students’ attitudes toward the teacher and peer feedback (Teng and Zhang, 2016), for example, openness to seeking help from instructors or peers when needed. This finding, which suggests that the intervention encouraged students to seek help and use feedback for improving writing outcomes, may be attributed to the way their teachers provided feedback (Lee, 2011; Hawe and Parr, 2014). The feedback process in this study, emphasizing self-regulation, differed from conventional feedback practice in that it points “forward to the students’ future writing and the development of his or her writing process” (Hyland and Hyland, 2006, p. 83), and benefited the treatment group students. Such SRL-based feedback practice, directed at triggering cognitive processing with an active response for deeper learning, encourages students to use the teacher’s feedback positively and creates a trusting environment for students to apply teachers’ feedback in a flexible way.

Moreover, the significant increase in interest enhancement suggests that students from the treatment group became more strategic, which increased their interest in learning. As Pintrich and Zusho (2002) claimed, motivational factors, interest, and value beliefs influence the development of self-regulated learning. Therefore, the students who are interested in an activity or task, and perceive it as valuable or important, are more likely to use self-regulatory strategies.

## Conclusion

In summary, this study provides empirical evidence for the sustained effect of teacher feedback on improving EFL learners’ writing outcomes and use of self-regulated writing strategies in a multiple-draft writing task. The results of the present study showed that the SRL-based feedback, implemented in an EFL writing class, enhanced students’ use of SRL writing strategies by substantially increasing goal-oriented monitoring, knowledge rehearsal, feedback handling and interest enhancement. SRL-based feedback appeared to be more effective with overall writing performance and the subcategories of organisation, vocabulary, and content than with language use.

As with all L2 writing feedback studies, this study is not without limitations. First, supplementary activities in the intervention may have mediated the effects of process-SR level feedback when compared to task-level feedback. As the present study is pedagogically driven, the intervention of SRL-based feedback was designed to include setting goals, keeping error logs and writing reflective journals. Although these activities were justified as necessary components of SRL-based feedback loop, further studies could exclude these activities to compare between the process-SR level and the task-level feedback. Moreover, the data included only students’ self-reported use of SRL writing strategies which may not be reliable. Further studies can collect qualitative data to examine how the SRL-based feedback loop influences students’ development of SRL writing strategies.

The current study, despite the noted limitations above, makes an important contribution to the literature on the effects of teacher feedback. It also provides an empirically based rationale for understanding the impact of feedback on L2 writing and L2 writers. First, the findings suggest that the SRL-based feedback loop can be an effective practice to enhance students’ writing performance and use of SRL writing strategies in the EFL context. Higher-order processes of self-regulation, described by Carless (2019) as products of reflection, may change writers’ performance in their next writing assignment, their repertoire of writing strategies, or some other aspects of the learning process which shape knowledge and beliefs. Second, the effectiveness of comprehensive feedback also depends on other mechanisms or circumstances. For example, in this study, the chance of revision and reflection allowed students to improve their writing quality by being more aware of problems and weaknesses in their texts.

## Data availability statement

The raw data supporting the conclusions of this article will be made available by the authors, without undue reservation.

## Ethics statement

The studies involving human participants were reviewed and approved by University of Auckland Human Participants Ethics Committee (Ref. 021440). The patients/participants provided their written informed consent to participate in this study.

## Author contributions

LY conceived of the initial idea, designed the study, collected and analyzed the data, and drafted the manuscript. YL and ZX shared the ideas for research design and revised the manuscript. YL proofread and finalized the manuscript for submission as the corresponding author. All authors contributed to the article and approved the submitted version.

## Appendix

### Appendix A: Sample feedback. Based on data from la Fata Almendral (2014)

Model of feedback for draft one at the process and self-regulation level in the treatment group.

**   The Essay of what I am writing about is the New York States role**
**in the American Revolutionary War.**

Model of feedback for draft two at the process and self-regulation level in the treatment group.

*   check the     reread and check the sentence*

*   capitalization   structure and meaning     check the word form*

**   The [Essay] [of what I am writing about is] the New York [States] role**

**in the American Revolutionary War.**

Model of feedback at the task level in the control group.

*   essay  that          is about         state’s*

**  The [Essay] of what I am writing [about is] the New York [States] role**

*give some information about New York’s role.*

*Then go into more detail in later paragraphs*
**in the American Revolutionary War.**
